# Methodological Frameworks and Dimensions to Be Taken Into Consideration in Digital Health Technology Assessment: Protocol for a Scoping Review

**DOI:** 10.2196/39905

**Published:** 2022-10-11

**Authors:** Joan Segur-Ferrer, Carolina Moltó-Puigmartí, Roland Pastells-Peiró, Rosa Maria Vivanco-Hidalgo

**Affiliations:** 1 Health Technology Assessment and Quality of Care Area Agency for Health Quality and Assessment of Catalonia Barcelona Spain

**Keywords:** digital health, eHealth, mobile health, artificial intelligence, framework, health technology assessment

## Abstract

**Background:**

Health technology assessment (HTA) is one of the main tools that health systems have to appraise evidence and determine the value of a given health technology. Although the existing HTA frameworks are useful tools for the evaluation of a wide range of health technologies, more and more experts, organizations across the world, and HTA agencies are highlighting the need to update or develop specific methodological frameworks for the evaluation of digital health technologies in order to take into account additional domains that cover these technologies’ intrinsic characteristics.

**Objective:**

The purpose of our scoping review is to identify the methodological frameworks that are used worldwide for the assessment of digital health technologies; determine what dimensions and aspects are being considered; and generate, through a thematic analysis, a proposal for a methodological framework that is based on the most frequently described dimensions in the literature.

**Methods:**

The scoping review will be performed in accordance with the guidelines established in the updated statement of the PRISMA-ScR (Preferred Reporting Items for Systematic Reviews and Meta-Analyses extension for Scoping Reviews). We will search for peer-reviewed and grey literature published between 2011 and the date of the search execution. The retrieved references will be reviewed in a single-blind manner by 2 independent authors, and their quality will be assessed by using the Critical Appraisal Skills Program tool. The ATLAS.ti software (Scientific Software Development GmbH) will be used for data extraction and to perform the thematic analysis.

**Results:**

The scoping review is currently (May 2022) in progress. It is expected to be completed in October 2022, and the final results of the research will be presented and published by November 2022.

**Conclusions:**

To our knowledge, no studies have been published to date that identify the existing methodological frameworks for digital HTA, determine which dimensions must be evaluated for correct decision-making, and serve as a basis for the development of a methodological framework of reference that health care systems can use to carry out this kind of assessment. This work is intended to address this knowledge gap of key relevance for the field of HTA.

**International Registered Report Identifier (IRRID):**

DERR1-10.2196/39905

## Introduction

### Background

European health systems, including the Spanish National Health System, face different challenges associated mainly with the progressive aging of the population [1-3]; the increasing prevalence of chronic conditions [2]; the growing need to medicalize citizens [1]; the rapid growth of health care expenditures, which are exceeding national incomes [1,4]; or the unequal distribution of health services throughout the territories [2,5]. Likewise, the health crisis caused by the SARS-CoV-2 (COVID-19) pandemic has increased the stress on health systems, challenging their sustainability and the values of universality, equity, and quality on which they are based [1,6-9]. Further, the COVID-19 pandemic has forced a hasty change from the face-to-face care model to a non–face-to-face model [7].

In this context, digital health, which is defined by the World Health Organization (WHO) as “the field of knowledge and practice associated with the development and use of digital technologies to improve health” [10] and by the European Commission as “the set of tools and services that use information and communication technologies to improve prevention, diagnosis, treatment, monitoring and management of health-related issues and to monitor and manage lifestyle-habits that impact health” [11], offers a unique opportunity to face these challenges and improve the accessibility, efficiency, sustainability, and quality of health systems [7,12].

The integration of digital health technologies (eg, mobile health [mHealth] apps, artificial intelligence [AI]–based solutions, etc) in health systems, however, entails certain challenges that hinder its implementation. Generally, these challenges are related to the rights of patients, the ownership of data, acceptance by users, the absence of adequate technological infrastructures, the literacy of professionals and patients, or the lack of robust evidence that makes the decision-making process difficult and can result in the development and reproduction of low-value technologies with a short, useful life span [7,10].

With regard to this last aspect, one of the main tools that the Spanish National Health System uses to generate evidence and determine the value of a given health technology is the health technology assessment (HTA) [13]. HTA, as well as its definition, has evolved since the 1980s, incorporating different dimensions in addition to safety, efficacy, effectiveness, and efficiency, such as the inclusion of the patient perspective, organizational aspects, or social impacts [6]. Currently, *health technology assessment* is defined as a “multidisciplinary process that uses explicit methods to determine the value of a health technology at different points in its life cycle,” and it is intended to inform decision-making processes in order to promote an equitable, efficient, and high-quality health system [13].

HTA is generally carried out by using specific methodological frameworks, such as the HTA Core Model of the European Network for Health Technology Assessment (EUnetHTA; known since 2022 as the *EUnetHTA 21 Consortium*) [14]. In Spain, HTA is performed by using the Guideline for the Development and Adaptation of Rapid Health Technology Assessment Reports of the Spanish Network of Agencies for Assessing National Health System Technologies and Performance, which was developed based on the HTA Core Model and other methodological frameworks [15]. Generally, these frameworks specify and standardize methods for evaluating the quality and value of health technologies, as well as the relevant information or elements that must be reported for a complete HTA. In this sense, the HTA Core Model 3.0 describes the following nine domains to be evaluated [14]: health problem and current use of technology, description and technical characteristics, safety, clinical effectiveness, costs and economic effectiveness, ethical analysis, organizational aspects, patient and social aspects, and legal aspects.

Although these frameworks are useful tools for the evaluation of a wide range of health technologies, more and more experts, organizations across the world (eg, the WHO), and HTA agencies (eg, National Institute for Health and Care Excellence [NICE], Canada’s Drug and Health Technology Agency, Finnish Coordinating Center for Health Technology Assessment [FinCCHTA], etc) are highlighting the need to update or develop specific methodological frameworks for the evaluation of digital health technologies that take into account additional domains (eg, interoperability, usability, etc) that cover these technologies’ intrinsic characteristics [7,10,16]. For this reason, some initiatives have emerged, such as the Evidence Standard Framework of the NICE [17] or the Digi-HTA Framework of the FinCCHTA [18]. However, most of these initiatives have some limitations, such as the development being conducted according to a specific socioeconomic or national context that hinders the transferability or applicability of the tool or framework to other countries, the specificity or exclusion of certain digital health technologies with limitations in their use, or the low evidence available in relation to the real usefulness of the methodological frameworks.

In this context, we intend to develop a scoping review with the aim of identifying the methodological frameworks that are used worldwide for the evaluation of digital health technologies; determining what dimensions and aspects are being considered; and generating, through a thematic analysis, a proposal for a methodological framework that is based on the most frequently described dimensions in the literature.

### Identifying the Research Questions

The scoping review will answer the following research questions:

- What methodological frameworks currently exist for digital HTA?
- What dimensions are being considered for the digital HTA?
- What dimensions are being described in more frequency in existing methodological frameworks?
- Are different dimensions being considered depending on whether the HTA is for a non–face-to-face care model of health care provision, a mobile device (mHealth), or a device that incorporates AI?

## Methods

### Overview of Methods for Conducting the Scoping Review

The scoping review of the available scientific literature will be carried out in accordance with the guidelines established in the updated statement of the PRISMA-ScR (Preferred Reporting Items for Systematic Reviews and Meta-Analyses extension for Scoping Reviews, see Multimedia Appendix 1) [19], with the aim of guaranteeing the transparency and reproducibility of the results.

Different experts in the fields of HTA and digital health technology participated in the planning of the study. Furthermore, they will be involved in its execution.

### Identifying Relevant Studies

The search strategy will be designed by an information specialist (RPP) and be based on the validated filter of Ayiku et al [20] for health apps; we will add the terms for concepts related to mHealth, remote care models, AI, digital health, methodological frameworks, and HTA. Taking into account the research questions, the initial search strategy for MEDLINE Ovid was designed by the information specialist (RPP) and peer-reviewed according to the Peer Review of Electronic Search Strategies Statement by JSF and CMP. The initial search strategy (Multimedia Appendix 2) will be exported to the following electronic databases: Ovid via MEDLINE, CINAHL Plus, Embase, Cochrane Library, Scopus, Web of Science, and TripDatabase. The characteristics of each database related to syntax, controlled vocabulary, and proximity operators will be taken into account. No time, language, or other filters will be used.

The identification of the studies will be complemented with a manual search that will be based on the references of the included studies, as well as the websites of the HTA agencies detected through the web pages of the EUnetHTA, the International Network for Agencies for Health Technology Assessment, and Health Technology Assessment International. Finally, a search will be carried out in Google Scholar, which will include the first 250 items to ensure that no relevant results are missed [21].

### Inclusion Criteria

The criteria for the selection of studies in the reference screening process will be based on the previously detailed research questions, and these criteria are described in Textbox 1, using the PICo-D (Population, Phenomenon of Interest, Context, and Design) format [22]. It should be noted that the PICo-D format has been used instead of the traditional PICO-D (Population, Intervention, Comparator, Outcomes, Design) format due to the qualitative nature of the research questions and the characteristics of the phenomenon of interest.

**Research questions in the PICo-D (Population, Phenomenon of Interest, Context, and Design) format (inclusion criteria).**

**Problem**

- Digital health technology assessment

**Phenomenon of interest**

- Specific methodological frameworks for the evaluation of digital health (with a special focus on mobile health, non–face-to-face models, and devices that incorporate artificial intelligence) that describe the domains that must be taken into account in this type of process, as well as the levels of evidence that should be considered for this process

**Context**

- Health technology assessment

**Design**

- Methodological guidelines and frameworks, scoping reviews, systematic reviews, consensus documents, and qualitative studies

In the study selection process, studies published before 2011, studies that do not describe dimensions or evaluation criteria, studies that are based on methodological frameworks that are not intended for this purpose (eg, EUnetHTA Core Model 3.0), comments, editorials, letters, and conference abstracts will be excluded. Likewise, methodological frameworks or tools that focus on the evaluation of digital health technologies by users (eg, user version of the Mobile App Rating Scale) and documents in languages other than English, Spanish, or Catalan will also be excluded. Nevertheless, in the case that we identify any methodological frameworks written in languages other than those mentioned above, the authors of those documents will be contacted to confirm the absence of an English version. Additionally, the translation of the documents will be considered.

All identified references will be imported into the EndNote bibliographic citation manager (version 20.2.1; Clarivate) [23], and duplicates will be removed according to the guidelines of Bramer et al [24].

The selection of studies will be carried out in 2 different phases. The first one will be the selection of studies via a single-blind peer review of the titles and abstracts of the references identified in the bibliographic search. This will be conducted by authors CMP and JSF. The second one will be a full-text, single-blind review of the studies included in the first phase, and this will be carried out by the same authors (CMP and JSF) in accordance with the selection criteria detailed above.

The quality of the evidence will be assessed by CMP and JSF using the Critical Appraisal Skills Program tool [25]. However, it should be noted that this tool cannot be used to obtain an overall score of the quality of the studies. Therefore, no references will be excluded due to their quality.

### Charting the Data

After the selection of the articles, the data of the included studies will be extracted. This task will be carried out by 3 reviewers (CMP, RPP, and JSF) using the web and desktop versions of the ATLAS.ti software (version 22.0; Scientific Software Development GmbH) [26] and the data extraction sheets that were designed ad hoc for this purpose according to the recommendations of the *Cochrane Handbook for Systematic Reviews of Interventions* [27]. The data to be extracted through the tables will be authors, publication dates, methodological framework/tool names, HTA agencies, countries, study designs, technology characteristics, the number of dimensions and criteria, dimensions, and detailed criteria.

For cases of discrepancies in either of the two processes (selection of studies or data extraction), a consensus will be reached among 3 reviewers (CMP, RPP, and JSF). If a discrepancy remains, a fourth reviewer (RVH) will be consulted.

### Collecting, Summarizing, and Reporting the Results

The evidence will be analyzed by using 2 approaches. First, a descriptive analysis will be carried out to evaluate and report the existing methodological frameworks and their characteristics. Second, a thematic analysis will be carried out according to the following three phases, which are described by Thomas and Harden [28], to identify HTA dimensions for digital health technologies: (1) line-by-line text coding, (2) the development of descriptive topics, and (3) the generation of analytical themes. Both analyses will be executed by three of the authors (CMP, RPP, and JSF) using the web and desktop versions of the ATLAS.ti software (version 22.0) [26].

The synthesis of the evidence will be carried out in a narrative manner, taking into account the selection criteria and the research questions detailed above.

Dimensions identified from systematic reviews that derived data from primary studies that are also identified in our systematic search will only be counted once in order to avoid the duplication of data and the risk of bias.

### Ethical Considerations

No ethical board approval is necessary to conduct this scoping review.

## Results

The scoping review is currently (May 2022) in progress. It is expected to be completed in October 2022, and the final results of the research will be presented and published by November 2022.

A dissemination plan has been developed to share the knowledge generated from the scoping review. Specifically, the results obtained from our work will be openly published in a scientific paper by March 2023, and they will also be presented at a national congress and an international congress. Furthermore, the results will be shared with the Spanish Ministry of Health, other relevant Spanish health care stakeholders, and national and international HTA agencies via direct emails and webinars.

## Discussion

### Principal Findings

Our scoping review is expected to identify and evaluate the existing frameworks for digital HTA and generate, through a thematic analysis, a proposal for a methodological framework that is based on the most frequently described dimensions in the literature. Although there are not many published frameworks that specifically address the assessment of digital health technologies, it is expected that considerable differences will be found among them (eg, in terms of the dimensions considered or the kinds of digital health technology addressed). Besides, additional domains that can be compared to those of conventional HTA methodological frameworks (eg, HTA Core Model) will probably be found. Finally, this work can be useful for the HTA field, as it will outline the main additional dimensions that should be considered for digital HTA and propose a framework that covers the intrinsic characteristics of digital health technologies [7,10,16].

### Comparison With Previous Works

There are some publications that focus on analyzing—through qualitative studies, narrative reviews, or systematic reviews—what information related to the dimensions of the EUnetHTA HTA Core Model are reported by studies on digital health, what methodological frameworks and tools exist for the evaluation of digital health technologies, and what dimensions are considered by these frameworks. However, none of the articles identified through the preliminary literature search, which was done before the development of this protocol, addresses the same research questions from the perspective of HTA. For example, a review by Moshi et al [29] analyzed 45 tools for the evaluation of mobile apps, regardless of their intended audience, and a review by von Huben et al [30] analyzed the degree to which such tools cover the evaluation domains of the EUnetHTA HTA Core Model 3.0.

### Strengths and Limitations

There are 2 main strengths to our study. First, different experts in the fields of HTA and digital health technology will participate in the planning and development of the study. Second, the validated filter of Ayiku et al [20] has been used to develop the search strategy. The main limitation of our study is the exclusion of frameworks published in languages other than English, Spanish, or Catalan. Another limitation is the use of controlled vocabulary that is not suited to the current state of knowledge in the digital health field.

### Future Directions

According to the WHO [10], there is low evidence available in relation to the real usefulness of the existing methodological frameworks for digital HTA. Future work will be conducted to explore the utility of the methodological framework that will be developed based on our scoping review and compare it to existing frameworks.

### Conclusions

To our knowledge, no study has been published so far with the aim of identifying the existing methodological frameworks for digital HTA, determining which dimensions must be evaluated for correct decision-making from the HTA perspective, and serving as a basis for the development of a methodological framework of reference that health care systems can use to carry out this kind of assessment. This work is intended to address this knowledge gap and may be useful in the field of HTA.

## Appendix

Multimedia Appendix 1 PRISMA-P (Preferred Reporting Items for Systematic Review and Meta-Analysis Protocols) checklist.

Multimedia Appendix 2 Search strategy.

## Abbreviations

AI: artificial intelligence
EUnetHTA: European Network for Health Technology Assessment
FinCCHTA: Finnish Coordinating Center for Health Technology Assessment
HTA: health technology assessment
mHealth: mobile health
NICE: National Institute for Health and Care Excellence
PICO-D: Population, Intervention, Comparator, Outcomes, Design
PICo-D: Population, Phenomenon of Interest, Context, and Design
PRISMA-ScR: Preferred Reporting Items for Systematic Reviews and Meta-Analyses extension for Scoping Reviews
WHO: World Health Organization
