# Supplementary material for: Methodological Frameworks and Dimensions to Be Taken Into Consideration in Digital Health Technology Assessment: Protocol for a Scoping Review
Source: JMIR Res Protoc. 2022 Oct 11;11(10):e39905. doi: 10.2196/39905 (PMC9597420; doi:10.2196/39905)
Supplement: Multimedia Appendix 2 [file resprot_v11i10e39905_app2.doc]

## Multimedia Appendix 2. Search strategy

| # | Search |
| --- | --- |
|  |  |
| 1 | Mobile Applications/ |
| 2 | exp Internet/ |
| 3 | exp Cell Phone/ |
| 4 | exp Computers, Handheld/ |
| 5 | Medical Informatics Applications/ |
| 6 | Therapy, Computer-Assisted/ |
| 7 | (app or apps).ti,ab. |
| 8 | (online or web or internet or digital*).ti. |
| 9 | ((online or web or internet or digital*) adj3 (based or application* or intervention* or program* or therap*)).ab. |
| 10 | (phone* or telephone* or smartphone* or cellphone* or smartwatch*).ti. |
| 11 | ((phone* or telephone* or smartphone* or cellphone* or smartwatch*) adj3 (based or application* or intervention* or program* or therap*)).ab. |
| 12 | (mobile health or mhealth or m-health or ehealth or digital health or e-health or emental or e-mental).ti. |
| 13 | ((mobile health or mhealth or m-health or ehealth or digital health or e-health or emental or e-mental) adj3 (based or application* or intervention* or program* or therap*)).ab. |
| 14 | (mobile* adj3 (based or application* or intervention* or device* or technolog*)).ti,ab. |
| 15 | or/1-14 |
| 16 | exp Wearable Electronic Devices/ |
| 17 | (Wearable* or ((Fitness or Activity) adj3 Track*) or Smartglasses or "Smart Glasses" or (("Head Mounted" or Headmounted or "Head Worn" or "Head Up") adj3 Display*) or "Google Glasses" or SMS or telemed* or telecomm* or "text messag*" or "short message service*" or tablet* or "electronic device*").ab,ti. |
| 18 | exp Telemedicine/ |
| 19 | exp Monitoring, Physiologic/ |
| 20 | exp Machine Learning/ |
| 21 | exp Artificial Intelligence/ |
| 22 | (Telerehabilitation or Teleradiology or monitoring or "IoT" or "Machine Learning" or "Deep learning" or Telerobotics or Robotics or Robotic or ((Artificial or Computational) and Intelligence)).ab,ti. |
| 23 | or/16-22 |
| 24 | 15 or 23 |
| 25 | exp Consensus/ |
| 26 | Guidelines as Topic/ |
| 27 | exp Practice Guidelines as Topic/ |
| 28 | Health Planning Guidelines/ |
| 29 | guideline.pt. |
| 30 | practice guideline.pt. |
| 31 | (position statement* or policy statement* or consensus or framework*).ab,ti. |
| 32 | (standards or guideline or guidelines or recommendat*).ab,ti. |
| 33 | or/25-32 |
| 34 | exp Technology Assessment, Biomedical/ |
| 35 | (HTA or ("Health Technology" adj3 (Assessment or Evaluation or Appraisal or Validation))).ab,ti. |
| 36 | 34 or 35 |
| 37 | 24 and 33 and 36 |
